# A Case of Vesicular Mole

**Published:** 1880-01

**Authors:** A. S. Coe

**Affiliations:** Oswego, N. Y.


### A CASE OF VESICULAR MOLE.

REPORTED BY A. S. COE, M. D., OSWEGO, N. Y.

Mrs. D., aged forty-two years, living in the country eight miles from Oswego, consulted me Nov., 1876. She brought with her the remains of four or five cysts preserved in alcohol; they were shriveled and corrugated by the action of the alcohol, and of a yellowish white color.

In 1866 she was delivered, with forceps, of a still-born child, after a protracted labor of four days and five nights. June 10th, 1872, she had an abortion at the commencement of the third month of pregnancy, but clots only came away as far as she observed.

In ten days from the beginning of the attack, the discharge had nearly ceased, and she resumed her household duties.

Her catamenia appeared twenty-five days after her recovery, and it has returned up to the present time, with a tolerable degree of regularity; the menstrual flow has been somewhat more profuse and protracted than before the abortion, but not to such an extent until quite recently as to materially impair her health or strength. Only once, September, 1876, had the hemorrhage been very profuse and alarming, at which time it continued excessive for twelve hours. Two years after the abortion she observed a watery discharge subsequent to each menstrual flow, and lasting about three days after which she noticed small cysts with the watery discharge, similar, but rather smaller in size, to those she brought for me to examine.

The cysts became larger in size after each successive menstrual period until they attained their present growth. They varied from six to fifty in number; she also observed that her abdomen was enlarging. The uterus, it was found, upon examination, had enlarged to the size of the third month of pregnancy, and apparently symmetrical in its growth—the cavity and neck measuring seven inches, and the sound deflecting to the right; the os was slightly patulous. Upon withdrawing the sound, two cysts about one and a half inches in length, oblong, oval in shape, and distended with a semi-gelatinous fluid, resembling the gelatin of Wharton, followed. Ergot was prescribed. Nothing more was seen of the patient until the last of February following. The ergot had produced no effect; she had continued to discharge the vesicles after each menstrual period as before; abdomen had not increased in size, and her health was about the same.

She had become much alarmed, and urged that some attempt should be made to remove the contents of the womb. Dr. Dewitt was called in consultation; the neck of the womb was dilated and an exploration made. Nothing was found save one cyst, which had adhered to the inner end of a sponge tent and came away with it.

Menstruation continued with the same degree of regularity, discharging the usual and varying amount of cysts, until July, 1878, when she had an attack of rather profuse menorrhagia, lasting six hours. In November following she had an entire suppression of the menses, which continued four months. During the time of suppression she discharged no cysts, nor did her womb materially enlarge. In March, 1879, she again menstruated excessively for a period of two days, but no more than the average number of cysts came away. She again menstruated three weeks afterwards and flowed but little, but discharged a large number of cysts, which discharge was prolonged several days beyond the usual time; they varied more in size and seemed more pliable. Her general health had continued tolerably good up to this time, save some swelling of the ankles, slight shortness of breath and palpitation of the heart at times. She has enjoyed a fair degree of health up to the present.

She is now very irregular, her periods varying from two to three months apart. Since August last no more cysts were discharged until October, the last time she came under observation. Her uterus had enlarged to the size of the fourth month of pregnancy. Her general health had improved.

The above history presents some noticeable features unusual in the development of hydatiform mole: (1.) Slow growth and length of time from the abortion before the symptoms attracted attention. (2.) The discharge of cysts after each menstrual period, thereby preventing a rapid enlargement of the uterus; and it seems that the rapidity of growth and fecundity of the cysts depended greatly upon the menstrual molimen, as there was no increased enlargement of the uterus, or a greater number of cysts discharged, even after a suspension of the menses for four months; in fact the uterus seemed to enlarge more when the menses were the most active, and the intervals shortest. (3.) Absence of frequent and dangerous hemorrhages. (4.) The length of time which the disease has lasted (now over seven years) without seriously impairing the health of the patient. She has been interrupted only by brief intervals in pursuing her household duties, at the same time taking care of a small dairy. Dr. J. W. Underhill, in a monograph published in the *Obstetric Gazette*, Cincinnati, January, 1879, upon hydatiform mole, says: "It is rare that the hydatiform mole is retained longer than the sixth month; but exceptional cases occur in which its existence is prolonged to the usual period of normal pregnancy, or even much longer. An instance is given of its continuance during the ordinary term of gestation in Braithwait's Retrospect." He quotes Montgomery, who refers to Madam Boivin's work, page 74, which contains a table showing the number of months intervening in thirty-two cases, between the commencement of pregnancy and the expulsion of the vesicular mole, from which it appears that while in some instances they were expelled at three months, and in one case not until fourteen months, the average period being between six and seven months. He says: "Instances of much longer delay in their expulsion are given by others." Morgagne says: "Nor are examples wanting of a long-continued dropsy solved by a very great number of hydatids discharged from the uterus." Lassions speaks of a widow who had the abdomen enlarged more than five years before her death; on examination the uterus was found extended by hydatids. Percy, Litre, Jolly, Baudelocque and Madam Boivin relate instances of the expulsion of hydatids from the uterus at ten, eleven, twelve and a half, and fourteen months after conception, and Dr. Ryan says he knew a case of hydatids continue fourteen years, after which time several pints were discharged mixed with purulent matter. One of the cases above quoted differs from nearly all of this form of pregnancy, in the fact that the size of the abdomen was no greater than if the gestation had been normal.

Dr. R. A. Cleeman, of Philadelphia, reported in the *American Journal of Obstetrics*, May, 1875, a case in which, at the fourth month, the abdomen was as large as at the seventh month of pregnancy. Dr. P. S. Murphy, of Washington, D. C., reports a case in the *Obstetric Gazette*, July, 1878, where at the fourth month the uterus had attained the size of six months' natural gestation. These two cases are confirmatory of what is generally observed as to the rapid enlargement of the uterus to a capacity disproportionate to the supposed stage of pregnancy." The instances quoted above show that the usual course of the abnormal growth is a rapid development to a size sufficient to stimulate the uterine fibres to contraction, thereby often expelling the mole *en masse.*

This case is of unusual interest not only from the slow growth of the mole, and discharge of the cysts after each monthly period, but from the fact that she is near her menopause, and also that the disease seems to be partially held in abeyance during intervals of suppression.

